# Improving Soil Quality and Potato Productivity with Manure and High-Residue Cover Crops in Eastern Canada

**DOI:** 10.3390/plants10071436

**Published:** 2021-07-14

**Authors:** Judith Nyiraneza, Dahu Chen, Tandra Fraser, Louis-Pierre Comeau

**Affiliations:** 1 Charlottetown Research and Development Centre, Agriculture and Agri-Food Canada, 440 University Avenue, Charlottetown, PE C1A 4N6, Canada; tandra.fraser@agr.gc.ca; 2 Fredericton Research and Development Centre, Agriculture and Agri-Food Canada, 850 Lincoln Road, P.O. Box 20280, Fredericton, NB E3B 4Z7, Canada; chen.dahu@agr.gc.ca (D.C.); louis-pierre.comeau@agr.gc.ca (L.-P.C.)

**Keywords:** cover crop, grasses, legumes, manure, potato, root-lesion nematodes, *Verticillium dahliae*

## Abstract

Under intensive low residue agricultural systems, such as those involving potato (*Solanum tuberosum* L.)-based systems, stagnant crop yields and declining soil health and environmental quality are common issues. This study evaluated the effects of pen-pack cow (*Bos Taurus*) manure application (20 Mg·ha^−1^) and cover crops on nitrate dynamics and soil N supply capacity, subsequent potato yield, selected soil properties, and soil-borne disease. Eight cover crops were tested and included grasses, legumes, or a mixture of legumes and grasses, with red clover (*Trifolium pratense* L.) used as a control. Forage pearl millet (*Pennisetum glaucum* L.) was associated with highest dry matter. On average, red clover had 88% higher total N accumulation than the treatments mixing grasses and legumes, and the former was associated with higher soil nitrate in fall before residue incorporation and overwinter, but this was not translated into increased potato yields. Pearl millet and sorghum sudangrass (*Sorghum bicolor* × *sorghum bicolor* var. Sudanese) were associated with lower soil nitrate in comparison to red clover while being associated with higher total potato yield and lower numerical value of root-lesion nematodes (*Pratylenchus penetrans*), although this was not statistically significant at 5% probability level. Manure incorporation increased total and marketable yield by 28% and 26%, respectively, and increased soil N supply capacity by an average of 44%. Carbon dioxide released after a short incubation as a proxy of soil microbial respiration increased by an average of 27% with manure application. Our study quantified the positive effect of manure application and high-residue cover crops on soil quality and potato yield for the province of Prince Edward Island.

## 1. Introduction

Potato is the third most consumed food crop worldwide [1] and is the most important cash crop in Prince Edward Island (PEI), representing around 25% of Canadian potato production [2]. Potato yields have been stagnant in PEI for over a decade [3], probably due to a combination of factors such as drought spells, soil erosion, accelerated soil organic matter mineralization, and increasing disease pressure. Soil organic matter depletion and declining soil aggregation are common under crops requiring intensive and frequent soil tillage such as potato-based systems [4,5]. Additionally, due to relatively low N use efficiency of potato, specifically under wet climate characterized by high rainfall after potato harvest [6,7], potato cropping regions have been reported to contribute excess N to surface and underground waters [8,9]. Prince Edward Island is the only Canadian province with total reliance on groundwater for its drinking water, and studies have been conducted to identify the source and timing of nitrate loadings. It was demonstrated that nitrification occurs year-round, with nitrate produced in winter contributing significantly to total nitrate loading [10,11] when N mineralization is coupled with excessive moisture. Therefore, strategies to increase or maintain soil and potato productivity while reducing nitrate loading during wet months are needed. Organic amendments such as manure incorporation and cover crops can help to enhance potato and soil productivity as well as N use efficiency [12].

Manure represents a mean to increase potato production and to enhance physical and chemical soil properties and N use efficiency [4,12,13,14]. An application of 4 Mg·ha^−1^ of fresh broiler poultry (*Gallus gallus domesticus*) manure in New Brunswick, Canada, was associated with marketable potato yield increases ranging from 19% to 34% and increased macronutrient concentrations, soil microbial respiration, infiltration rate, and earthworm abundance [14]. Farmyard cattle manure applied at a rate ranging from 0 to 100 kg·N·ha^−1^ was associated with marketable yield increase of 25.1% in Italy [15] and increased N use efficiency but did not increase N leaching risk. Increased N use efficiency and potato yield were observed in another study in Michigan when manure was combined with mineral N fertilizer [12]. Application of manure and compost less frequently than annually was reported to enhance soil aggregation and soil organic carbon in potato-based systems [4].

Where manure is not available, introducing cover crops into potato production systems could enhance soil health and potato productivity. Reducing our reliance on fertilizer N through improved exploitation of symbiotic N_2_ fixation would be a good contribution to sustainability and resource use efficiency [16]. Crop yield, N uptake, and N mineralization were reported to increase following a legume cover crop, compared to a grass [17]. When managing legume cover crops, the main challenge is to synchronize N release with subsequent crop N demand. In PEI, the conventional crop rotation after potato harvest includes barley (*Hordeum vulgare* L.) underseeded with red clover, followed by a pure stand of clover in the second year. Due to a short growing season and a wet spring, growers prefer to plow in fall, which enhances the risk of nitrate leaching over winter and during snowmelt in spring [11,18]. Compared to red clover, including sorghum sudangrass was associated with lower nitrate leaching and increased potato yield in a study conducted in PEI [19].

Mixing legumes with grasses can increase soil nutrient recycling due to their differences in root depth and their growth pattern across the season. A dynamic relationship between legumes and grass exists, with grasses stimulating N fixation by legumes and N uptake by grass, decreasing the inhibitory effect of soil N on biological N fixation [16,20]. Combining grass’ N scavenging ability with legumes’ biological fixation capability modifies the C:N ratio in comparison with a pure stand of a grass and slows down the fast N mineralization from a legume in monoculture [21]. A grass and legume mixture demonstrated the potential to increase N use efficiency and to reduce leaching compared to a pure legume. Grass and legume together resulted in higher dry matter per unit area than respective monoculture [22,23,24,25]. Compared to a grass in monoculture, increased N concentration and lower C:N ratio were reported in a grass grown with legume [23].

Diversifying cover crops in potato-based systems is an efficient means to break disease cycles, which can contribute to increased potato yields. In PEI, the major disease limiting potato yield is potato early dying complex (PED), which is primarily caused by *V. dahliae* and is exacerbated by root-lesion nematode. Potato early dying complex could contribute to potato yield declines of up to 50% [26,27]. Forage legumes such as red clover were reported to host root-lesion nematodes [28,29], and alternative non-host rotational crops need to be explored. Forage pearl millet prior to growing potatoes was reported to be effective in suppressing root-lesion nematodes [30,31,32,33]. Certain green manures are associated with biofumigant properties, which help in reducing soil-borne disease. Sorghum sudangrass produces cyanogenic glucosides harmful to crop pests. In a 2-year study comparing sudangrass, winter pea (*Pisum sativum* L.), rapeseed (*Brassica napus* L.), rye (*Secale cereal* L.), oat (*Avena sativa* L.), and maize (*Zea mays* L.), researchers observed 30% to 80% *Verticillium* reduction on the potato crop following sudangrass and significantly greater yield than with other preceding crops [34]. Similar results were reported in another study [35]. Small potato yield increases and a reduction of *V. dahliae* density and wilt severity following one application of sorghum sudangrass, winter pea and broccoli (*Brassica oleracea* var. italica) were reported [36].

Breaking soil-borne disease with diversified cover cropping, such as mixing legumes with grasses to reduce N carryover prior to growing potatoes and incorporating animal manures, are strategies to enhance potato and soil productivities. The objectives of this study were to assess the effects of diversified cover crops with and without pen-pack cow manure on nitrate dynamics during the growing season and soil N supply capacity during subsequent potato phase, potato yield and quality, soil properties, and population density of root-lesion nematodes and *V. dahliae*.

## 2. Materials and Methods

### 2.1. Site Characteristics and Treatments

The field trial was conducted at Agriculture and Agri-Food Canada’s Harrington research farm (46°20′37.020″ N, 63°10′11.050″ W), PEI, Canada in 2017 and 2018. The experimental design was a split-plot design during the cover crop phase, with the main factor being manure (cow manure) and the subplot being different cover crops. The subplot was 26 m long and 8 m wide. Red clover, as the traditional cover crop in potato production, was compared with sorghum sudangrass, pearl millet, and different mixtures of crops: alfalfa (*Medicago Sativa*) and orchardgrass (*Dactylis glomerata*); forage sorghum followed by brown mustard (*Brassica juncea*); common ryegrass (*Lolium perenne*) and common vetch (*Vicia sativa*) and crimson clover (*Trifolium incarnatum*); sorghum sudangrass and verticillium-resistant alfalfa; and winter rye (*Secale Cereale*) and hairy vetch (*Vicia vilosa*). 

The soil at this site is a Charlottetown fine sandy loam classified as Ferro-Humic Podzol in the Canadian soil classification system, which corresponds to Orthic Podzol in the FAO classification system. Prior to the establishment of the plots, four soil sub-samples were taken to a depth of 15 cm in each block using a Dutch auger for initial site characterization. Average soil organic carbon (SOC) across blocks was 1.50% and was analyzed using dry combustion on an elemental analyzer (Vario MAX Elementar Analyzer, Hanau, Germany). Soil pH (1:1 soil solution ratio; 10 g soil: 10 mL water, [37]) was 5.9. Macro nutrients were analyzed using Mehlich-3 [38] solution, and initial P, K, Ca and Mg were 412.5 mg P_2_O_5_ kg^−1^, 196.7 mg K_2_O kg^−1^, 718.5 mg Ca kg^−1^ and 78.6 mg Mg kg^−1^, respectively. The cation exchange capacity was 6.4 meq 100 g^−1^, and total base saturation was 72.7%. 

Manure was incorporated (20 Mg·ha^−1^ wet basis) five weeks prior to seeding cover crops. Manure analysis was performed using the dry ash method [39], and had 30% dry matter. The percentages of C, N, P, K, Ca, and Mg contents were 34.7%, 1.77%, 0.36%, 1.5%, 0.63%, and 0.27%, respectively which correspond to a supply of 106, 22, 90, 38, and 16 kg·ha^−1^ of N, P, K, Ca, and Mg, respectively. On 8 June 2017, after the land was plowed, seedbed was rolled before and after seeding the following cover crops: (i) a mixture of alfalfa and orchardgrass at 17 kg·ha^−1^; (ii) forage pearl millet (CFPM101) at 34 kg·ha^−1^; (iii) forage sorghum sudangrass at 36 kg·ha^−1^ left to grow for 10 weeks and plowed under and then seeded with brown mustard at 10 kg·ha^−1^; (iv) red clover at 22 kg·ha^−1^; (v) a mixture of ryegrass (at 3 kg·ha^−1^), common vetch at 35 kg·ha^−1^ and crimson clover at 12 kg·ha^−1^; (vi) sorghum sudangrass hybrid at 34 kg·ha^−1^; (vii) a mixture of sorghum sudangrass at 19 kg·ha^−1^ and verticillium-resistant alfalfa at 6 kg·ha^−1^; and (viii) a mixture of winter rye at 125 kg·ha^−1^ and hairy vetch at 40 kg·ha^−1^. Cover crops that did not receive manure were supplied with 38 kg·N, P_2_O_5_ and K_2_O kg·ha^−1^ of mineral fertilizer when a cover crop mixture included a legume, while the cover crops without the presence of a legume received 76 kg·N, P_2_O_5_, and K_2_O kg·ha^−1^ of mineral fertilizer. 

During the potato phase in 2018, the subplot was split in two halves, with one half receiving no N application and the other half receiving the recommended N fertilizer. Potatoes were planted on 30 and 31 May 2018, with seed spacing of 30.5 cm and row spacing of 91 cm. Therefore, the sub-subplot during potato phase had four potato rows and was 21 m long. Before planting, muriate of potassium (0-0-60) was broadcast in all plots at 150 kg K_2_O ha^−1^. The rest of the fertilizer was banded with potato planting by supplying 150 kg·N·ha^−1^ as ammonium nitrate, 200 kg P_2_O_5_ ha^−1^ and 50 kg K_2_O as potassium chloride, while the plots without N fertilizer received 200 kg P_2_O_5_ ha^−1^ and 50 kg K_2_O as potassium chloride. Moldboard plowing was performed in fall after cover crop incorporation, and two passes with a cultivator were implemented in spring prior to seeding potatoes. Potato hilling was carried out after potato emergence.

### 2.2. Cover Crop Sampling and Analyses

Six weeks after seeding of cover crops, all cover crops were mowed and flailed to control weeds. The red clover was mowed and flailed again at 120 days after planting, and all plots were mowed and flailed on 2 October 2017, prior to their incorporation. Before each cut, cover crop biomass was taken using one m^2^ to quantify dry matter biomass. Biomass harvested from 1 m^2^ was weighed, a subsample of at least 500 g was taken, and the rest of the biomass was returned to their respective plots. The subsample was dried at 60 °C until constant mass. The cover crop biomass was ground to pass through a 0.15-mm sieve and analyzed for total C and N as described above. Total dry matter biomass over the growing season was the sum of dry matter biomass obtained at each cut. Nitrogen and C accumulation was calculated by multiplying N and C concentration at different sampling times with dry matter biomass, and the total N and C accumulations was the sum obtained at different cuts.

### 2.3. Soil Sampling and Analyses

In fall 2017 before crop residue incorporation and in spring 2018, soil samples were taken in each experimental unit by mixing four to five subsamples using a Dutch auger at 0–15 cm and 15–30 cm soil depth. Soil samples taken in fall 2017 and 2018 were analyzed for nitrate, whereas those taken in spring 2018 were analyzed for nitrate, permanganate oxidizable carbon to analyze active carbon [40], and carbon dioxide (CO_2_) released following a 24-h incubation by rewetting dried soil using the capillary method described by Haney and Haney [41]. Carbon dioxide expressed as μg CO_2_-C g soil^−1^ day^−1^ was analyzed using a Li-COR gas analyzer (Li-Cor-Li-830-850, Li-COR Biosciences, Lincoln, Nebraska, US).

In addition, under the plots not receiving N fertilizer during the potato phase, monthly soil samplings (June, July, August, September) were taken at two depths (0–15 cm and 15–30 cm) during potato growth and analyzed for nitrate. Total organic C was analyzed on soil taken in fall after potato harvest to calculate SOC as described above. In fall 2017 prior to incorporating cover crops, surface hardness (soil compaction) was assessed using a penetrometer (Spectrum Technologies Inc. S/N 22244, Hoskin Scientific, Oakville, ON, Canada) inserted to 15 cm at three randomly selected places within each plot. In addition, a block of soil of 0.008 m^3^ (20 cm × 20 cm × 20 cm) was dug using a shovel, the soil was spread and sifted through fingers over a transparent plastic bag, and the total number of earthworms was counted. The density of total earthworms per sampled volume was extrapolated per m^2^ × 20 cm depth.

### 2.4. Soil Nitrate Dynamics over Winter Following Cover Crop Incorporation Measured Using Anion Exchange Membranes

Nitrate released during fall and winter following cover crop incorporation was measured using anion exchange membranes (AEMs). They were installed from 1 November 2017, to 24 April 2018. The preparation of AEMs followed the method described by Ziadi et al. [42]. They were gently inserted in the ground at 0–15 cm using a trowel, and the soil was firmly pressed to maintain a good contact between the AEMs and the soil. Two AEMs per experimental unit were attached to fishing lines, which in turn were attached to a flag to facilitate their retrieval. Following AEMs collection, attached soil particles were removed with distilled water, and AEMs were shaken for 2 h in a solution containing 1 mol·L^–1^ KCl to extract nitrate. Nitrate was quantified after filtration using a Lachat QuikChem 8500 (Lachat Instruments, Loveland, CO, USA). Nitrate fluxes from AEMs were expressed as μg NO_3_-N cm^–2^·d^–1^ concentrations divided by AEM surface area and by the time they were left in the field, expressed in days.

### 2.5. Potato Sampling and Harvest

Total N accumulations in the whole potato plants (vines and tubers) were quantified by sampling four potato plants before vine desiccation. Vines were cut at the soil level, and tubers were recovered using a hand fork. Vines were weighed and chopped, and a subsample of at least 500 g was taken. Tubers were first washed and weighed, and a subsample was taken by slicing six tubers using a French fry cutter. The subsample was weighed, and vine and tuber subsamples were dried in an oven at 60 °C until constant mass. The concentration of N was determined using the dry combustion method on an Elementar analyzer (Vario Max, Elementar Analyzer, Hanau, Germany). Nitrogen accumulation in vines and tubers was obtained by multiplying the N concentration by the respective dry matter, and the total N accumulation was the sum of N accumulation in vines and tubers.

Potato harvest was carried out on 22 October 2018. One internal row was used for tuber harvest, and tuber size categories were determined as follows: Culls: <3.8 cm; Can1-small: 3.8 to 5.1 cm; Can1: 5.1 to 8.9 cm; Can1-large: 8.9 to 11.4 cm; Jumbo: >11.4 cm. Marketable yield consisted of Can1 and Can1-large. Due to the drought spell in 2018, we observed tubers with jelly end incidence, which were not included in the marketable yield. The proportion of tuber number with jelly end over the total number was calculated. 

### 2.6. Verticillium DNA Extraction and qPCR Quantitation and Root-Lesion Nematode Quantification

The soil samples, taken in fall 2017 before plant tissue incorporation, in spring 2018 prior to seeding potato, and post-potato harvest in spring 2019 after overwintering, were used to quantify the *Verticillium* population density. Soil samples of approximately 500 g were taken from each plot and stored at 4 °C until processing. Soil was then well mixed, air-dried, and sieved to 2 mm, with triplicate subsamples of 0.25 g each extracted using E.Z.N.A.^®^ Soil DNA Kit (Omega Bio-tek; cat# D5625) according to manufacturer protocol. Extraction success and cross-contamination control was assessed by extraction of known clean, sterile soil with (positive control) and without (negative control) the addition of a few grains (~0.1 mg) of dried, powdered fungal material scraped from pure culture plates of each *Verticillium* target species. Total soil DNA extracted was typically on the order of 100 µg·mL^−1^ as measured by spectrophotometer (Eppendorf BioPhotometer).

Concentrations of *V. dahliae* and *V. albo-atrum* DNA were quantified by probe-based quantitative real-time PCR (qPCR). Primer and probe sequences targeted the *ef1α* gene of each species and were acquired from Sigma-Aldrich or Integrated DNA Technologies Inc. (IDT). Duplex qPCR thermocycling proceeded at 95 °C, once for 3 min, then for 40 cycles of 95 °C for 15 s and 64 °C for 60 s in PerfeCTa^®^ Multiplex qPCR ToughMix^®^ (Quantabio, Beverly, MA, USA; cat# 95147-01K); all primers at 0.3 µM, probes 0.2 µM and with 0.1 µg/µL BSA. All reactions were conducted in a qTOWER^3^-G thermocycler (Analytik Jena AG, Jena, Germany). Absolute concentrations of each species’ DNA were calculated by comparing to a standard curve of target DNA designed into a synthetic gBlocks™ DNA standard (IDT, Newark, NJ, USA) [43]. This DNA target is present as a single copy per genome, thus DNA concentration could be directly converted to estimated genomic DNA concentration in soil using the known genome size of *V. dahliae* at 36.5 fg/cell [44]. The specificity of the gBlocks™ DNA targets were tested in simplex and duplex reactions, and concentrations were confirmed to be consistent with those determined empirically with *Verticillium* DNA extracted from pure cultures. DNA was quantified in duplicate from independent qPCR mixes to confirm results and values of each qPCR were averaged together; final estimated *V. dahliae* and *V. albo-atrum* DNA concentrations from each soil sample were reported as the mean of the three soil extract subsamples (each with duplicated qPCR run) ± standard error. Soil sample processing, DNA extraction, and qPCR quantitation were conducted at Agricultural Certification Services Inc. (Fredericton, NB, Canada).

The 2019 soil samples were also used to quantify the root-lesion nematodes (*Pratylenchus* spp.) based on the Baermann pan extraction method at Agriculture & Food Laboratory Services in University of Guelph. Baermann pan method was used for extraction of nematodes from soil [45]. Briefly, soil sample was mixed thoroughly first, and 50 g of soil was placed on facial tissues in a pan. Water was added so there was a thin layer of water in the bottom of the pan. The pan was placed in a plastic bag for 7 days at room temperature. After 7 days, the free water in the pan was poured into a test tube and the nematodes allowed to settle to the bottom of the tube in a refrigerator for at least 3 h. A hollow bent glass pipette was used to remove water from the tube and leave approximately 3 mL of water in the bottom of the tube and then the remaining water was transferred into a counting dish. A few drops of 1% surfactant (Tween) was added and allowed nematodes to settle before counting. Plant parasitic nematodes were identified and counted under a dissecting microscope (Nikon SMZ1500). After extraction, the wet soil was oven-dried at 85 °C for at least 3 days before weighing. Result was reported as “# of nematode kg^−1^ of dry soil”.

### 2.7. Statistical Analyses

The statistical analyses were performed using the MIXED procedure of SAS [46]. Normality was tested using the Shapiro–Wilk test, and logarithmical transformation was carried out when needed. Treatments (cover crops), manure and nitrogen fertilizer were treated as fixed factors, whereas block and block × manure were treated as random factors. Multiple comparisons among treatment means were performed using the DIFF option in SAS at the 0.05 probability level.

## 3. Results

### 3.1. Air Temperature and Precipitation in 2017 and 2018

**Figure 1:**
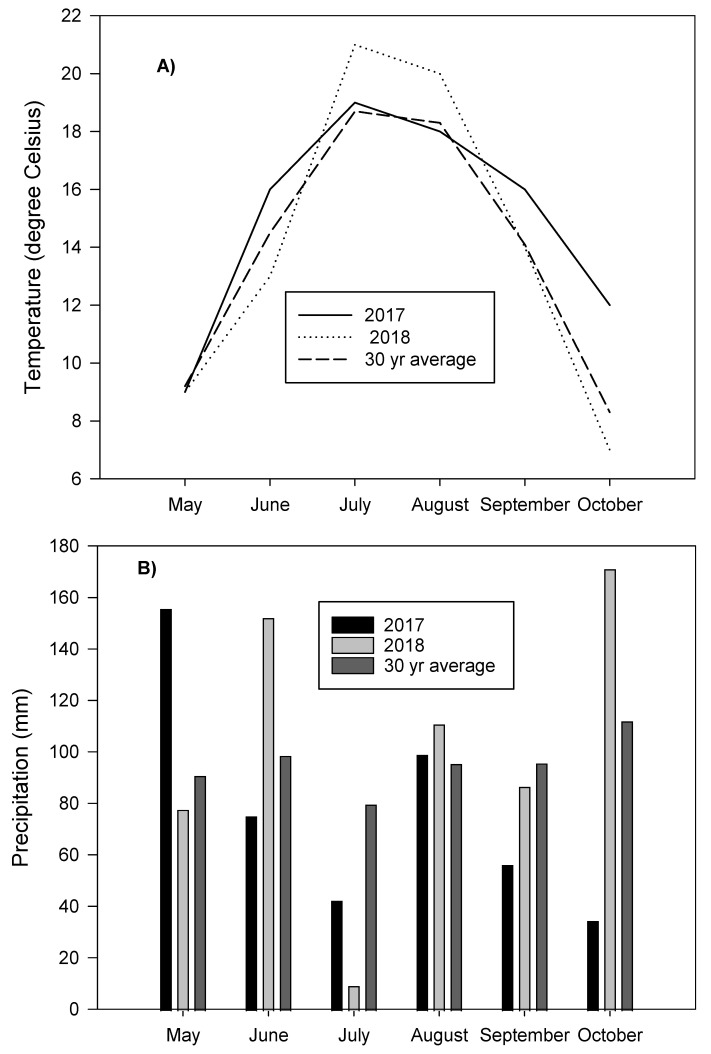
Mean monthly temperature (**A**) and total monthly precipitation (**B**) during the 2017 and 2018 growing seasons in comparison with the 30-year average (1981–2010) at Harrington Research Farm.

Compared to the 30-year average, mean monthly temperature in 2017 was higher in June, July, September, and October, whereas values were close to the 30-year average in May and August (Figure 1). In 2018, values were close to the 30-year average values in May and September, whereas they were slightly higher in July and August and lower in June and October. In 2017, May had precipitation 71% higher than the 30-year average, whereas it was lower in June, July, September, and October (Figure 1). In 2018, precipitation was higher than the 30-year average in June, August, and October, but was eight times lower in July and 17% and 10% lower in May and September, respectively (Figure 1). Water deficit in July during potato growth is expected to negatively impact potato yields as this is the time corresponding to tuber initiation and tuber growth. 

### 3.2. Effects of Cover Crop Treatment and Manure Application on Cover Crop Dry Matter Biomass, Carbon and Nitrogen Accumulations, and C:N Ratio

Cover crop dry matter biomass, C and N accumulations, and C:N ratio were not affected by manure application, but the effect of cover crop treatments was significant (Table 1). Dry matter biomass ranged from 2.7 to 6.2 Mg·ha^−1^, with forage pearl millet being associated with higher value, followed by red clover, ryegrass and common vetch, and crimson clover mixture and sorghum sudangrass (Table 1). The latter values were comparable to those recorded under forage sorghum followed by brown mustard, sorghum sudangrass and verticillium-resistant alfalfa mixture, and the lower values were associated with winter rye and hairy vetch. A similar trend was observed with total C accumulation (Table 1).

Total N accumulation in cover crops ranged from 67.7 to 145.6 kg·N·ha^−1^, with highest values associated with red clover, followed by forage pearl millet, which was comparable to forage sorghum, followed by brown mustard, ryegrass and common vetch and crimson clover mixture, and sorghum-sudangrass. Lower cover crop N accumulations values were associated with alfalfa and orchardgrass mixture, sorghum sudangrass and verticillium resistant alfalfa mixture, and winter rye and hairy vetch mixture (Table 1).

The C:N ratio of the cover crops at the time of their incorporation was higher and comparable among alfalfa and orchardgrass mixture, forage pearl millet, sorghum sudangrass, and sorghum sudangrass and verticillium-resistant alfalfa mixture, and were lowest with red clover (C:N = 14.4), followed by winter rye and hairy vetch mixture (C:N = 16.1) and the forage sorghum sudangrass followed by brown mustard (C:N = 17 for the mustard) (Table 1).

### 3.3. Effect of Manure and Cover Crop Treatments on Soil Nitrate in Fall Before Biomass Incorporation, and Over Winter as Measured With Anion Exchange Membranes during Potato Phase

**Figure 2:**
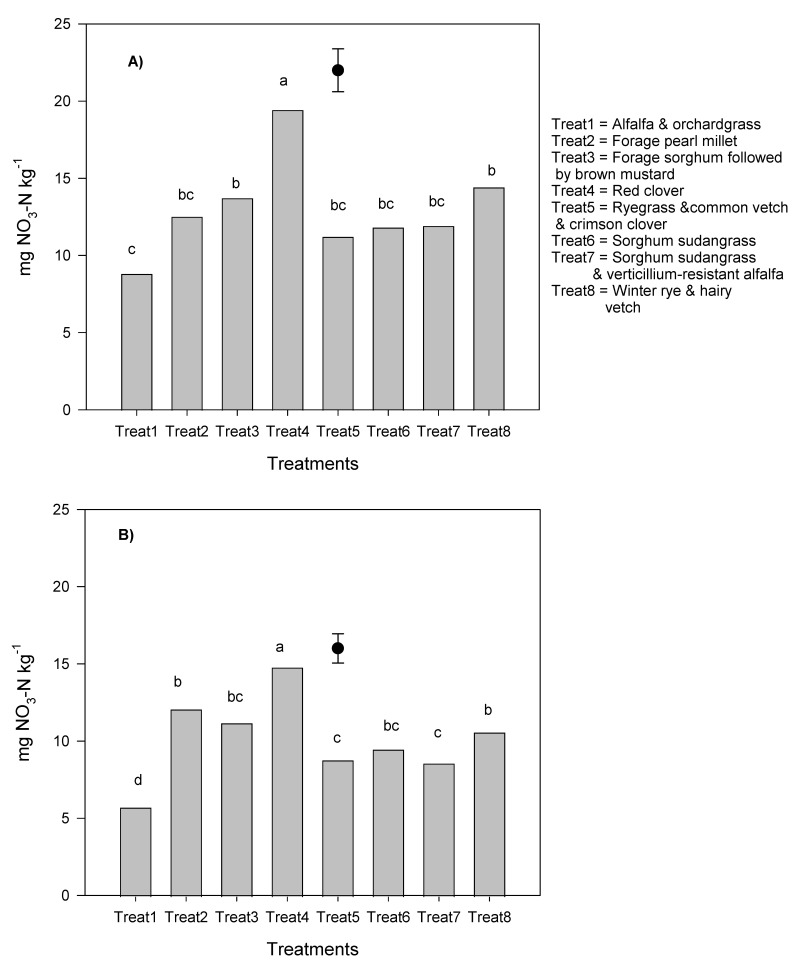
Nitrate contents in fall before cover crop incorporation at 0–15 cm (**A**) and 15–30 cm depth. (**B**) Values followed by different letters are statistically significant at 5% probability level. Vertical bars represent the standard error of the mean.

**Figure 3:**
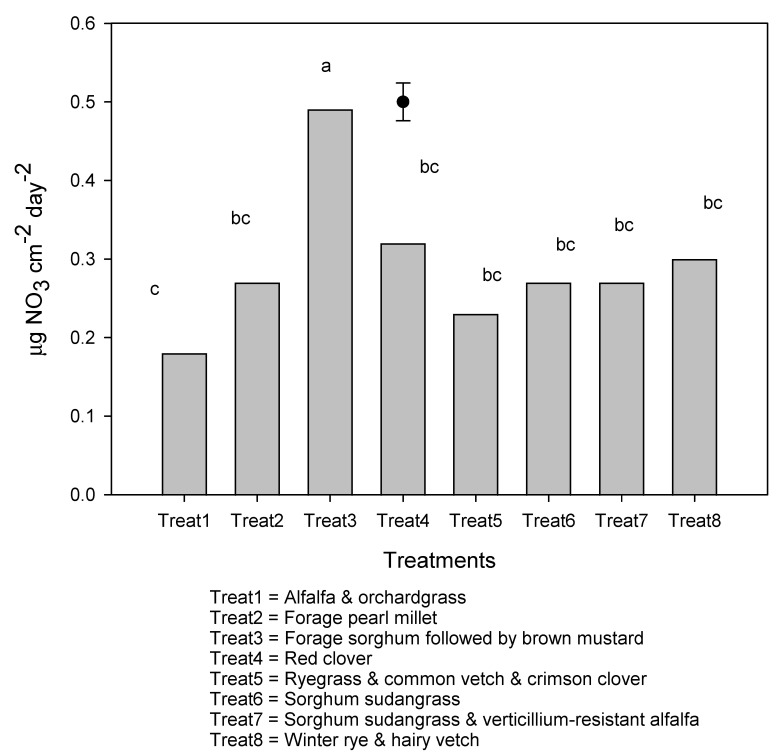
Nitrate contents over winter following cover crop incorporations captured by anion exchange membranes (AEMs). Values followed by different letters are statistically different at 5% probability levels. Vertical bar represents standard error of the mean. Anion exchange membranes were inserted on 1 November 2017, until 24 April 2018.

The effect of the manure was not significant on nitrate measured in fall before cover crop incorporation, but cover crop effect was significant (Figure 2A,B). Red clover was associated with higher nitrate than other treatments at 0–15 cm and 15–30 cm depth (Figure 2), and the lowest values were observed with alfalfa and orchardgrass. The effect of manure application was not significant on nitrate measured over winter using AEMs. However, cover crop effect was significant, with forage sorghum followed by brown mustard being associated with higher values and lowest treatment recorded under alfalfa and orchardgrass mixture, whereas all other treatments were comparable (Figure 3). Manure and cover crop effects were both significant on nitrate measured 7 months after cover crop incorporation (in May) at 0–15 cm depth and the cover crops, and the interactions between manure and cover crops were also significant at 15–30 cm depth (Table 2). Manure application was associated with higher nitrate values than without manure application at 0–15 cm, and higher values were observed with alfalfa and orchardgrass mixture and with ryegrass and common vetch and crimson clover mixture, which were comparable with red clover. Numerical lower values were associated with sorghum sudangrass and pearl millet (Table 2). The interaction between manure and cover crop treatments was significant on nitrate measured at 15–30 cm, with red clover showing higher values than other treatments when manure was not applied. When manure was applied, comparable and higher values were observed with red clover, forage sorghum, followed by brown mustard, and ryegrass and common vetch and crimson clover than other treatments. Manure and cover crop effects were not significant on nitrate measured over the potato growing season, and comparable values were observed among cover crop treatments.

### 3.4. Effects of Nitrogen Fertilizer Application, Manure and Cover Crop Treatments on Total and Marketable Yield, Specific Gravity, and on Jelly end Incidence

Total yield ranged from 23.9 to 29.9 Mg·ha^−1^, and manure increased total yield by 28%. Forage pearl millet, forage sorghum followed by brown mustard, and sorghum sudangrass were associated with higher total yields than red clover, rye grass and common vetch and crimson clover mixture, sorghum sudangrass and verticillium-resistant alfalfa (Table 3). A similar trend was observed with marketable yield.

**Figure 4:**
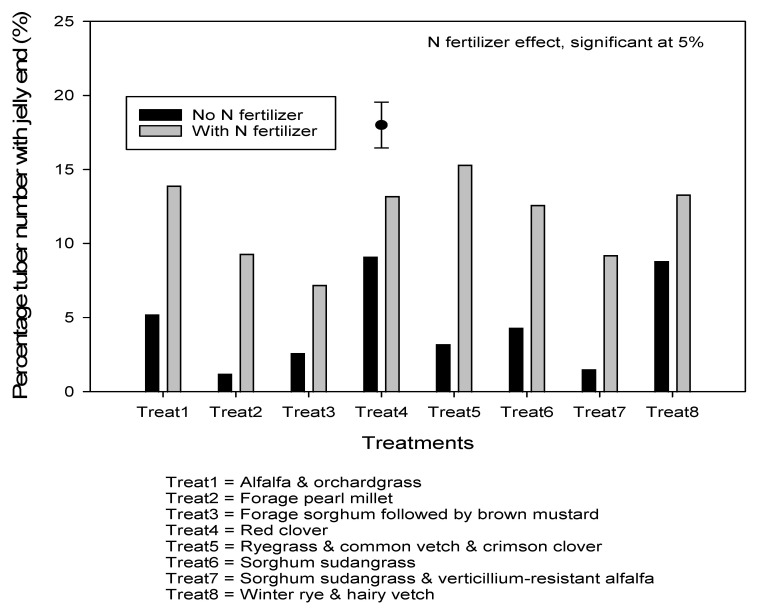
Percentage total tuber number with jelly end in response to N fertilizer. Vertical bar represents standard error of the mean.

Manure application increased marketable yield by 35% compared to no manure applied (Table 3). On average, marketable yield was 27% lower than total yield, most likely due to jelly end incidence. Nitrogen fertilizer effect was only significant on total yield, with higher values associated with N fertilizer application. Nitrogen fertilizer effect was also significant on specific gravity, with higher values associated with no N fertilizer application. Manure and cover crop treatments were not significant on percentage tuber number with jelly end, but N fertilizer effect was significant, with higher values associated with N fertilizer application (Figure 4).

### 3.5. Effect of Manure and Cover Crop Treatments on Active Carbon, Soil Respiration, Total Earthworm Abundance, Surface Hardness, and Soil N Supply Capacity

**Figure 5:**
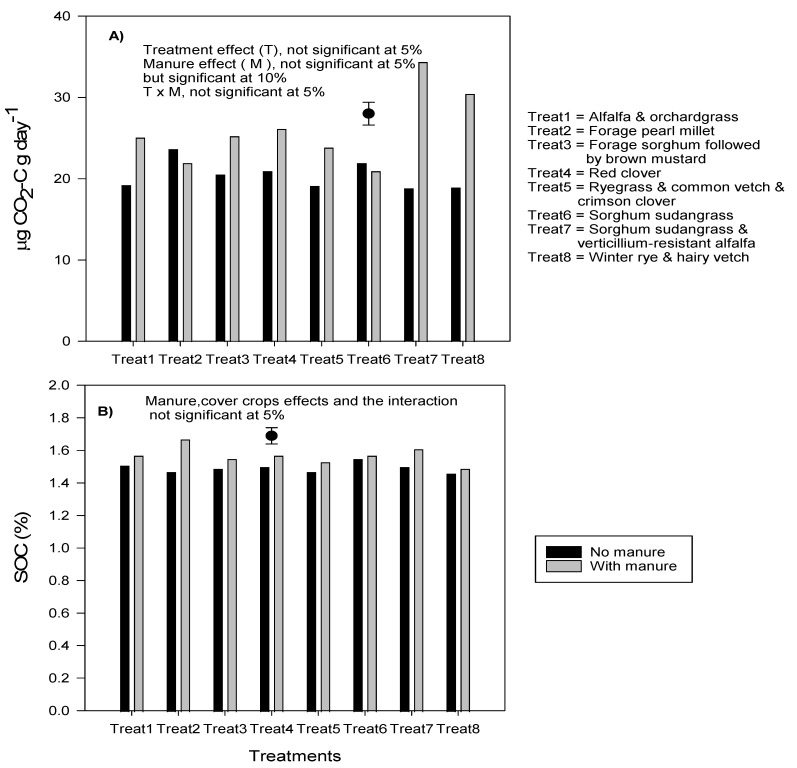
Carbon dioxide released after 24-h incubation on soil samples taken in spring before growing potatoes (**A**) and soil organic carbon (**B**) measured after potato harvest. Vertical bars represent standard error of the mean.

No effect of manure was observed on permanganate oxidazable carbon (active carbon), total earthworm or surface hardness (Table 4), but an average of 27% increase in soil respiration (flush of CO_2_ after rewetting a dried soil) was observed on soil with manure application in comparison with no manure application, although this was not significant at 5% probability level (Figure 5A). Cover crop treatment effect was not significant on any of the above-mentioned parameters. There was a trend toward higher SOC values with an average of 10% SOC increases with manure than without manure, even though manure effect was not statistically significant (Figure 5B).

**Figure 6:**
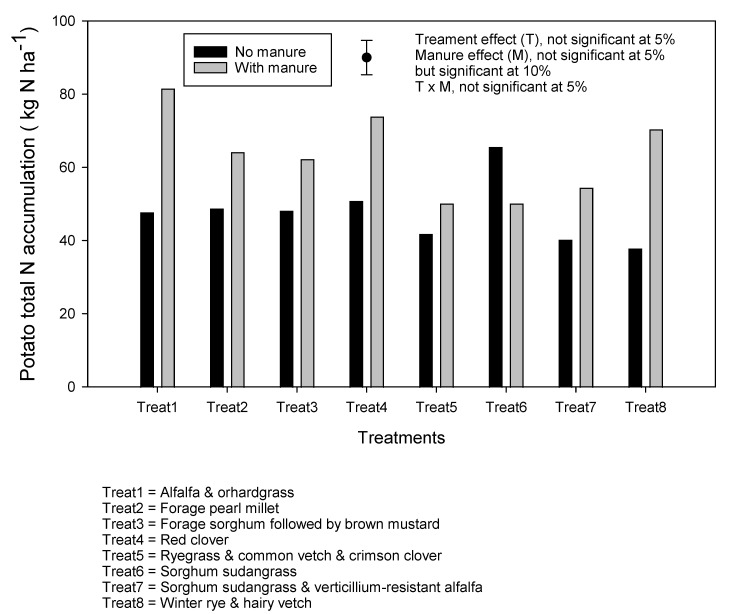
Total N accumulations in the potato plant (vine + tuber) with no N fertilizer application as a proxy of soil N supply. Vertical bar represents standard error of the mean.

Total N accumulation in plots without N fertilizer during potato phase was assessed to estimate soil N supply capacity. There was a clear trend towards increased soil N supply capacity with manure application, except for the sorghum sudangrass mixed with verticillium-resistant alfalfa. Manure increased soil N supply capacity by an average of 44%. Soil alone supplied 38–51 kg·N·ha^−1^ to potato crops without manure application and 44–81 kg·N·ha^−1^ when manure was applied. The cover crop treatment effect was not significant (Figure 6).

### 3.6. Effects of Manure and Cover Crop Treatments on Population Density of Verticillium and Root-Lesion Nematodes

*V.dahliae* was detected in all samples collected in 2017, 2018, and 2019, but *V. albo-atrum* was not. The effect of the manure application on *V. dahliae* population density was only detected in fall 2017 among the three sample years, with manure application significantly reducing the population density compared to no manure application (Table 5). No significant differences were detected among the cover crop treatments for the *V. dahliae*, although numerically lower values of *V. dahliae* were consistently observed with ryegrass and common vetch and crimson clover mixture and forage sorghum followed by brown mustard (Table 5). Root-lesion nematode densities were not determined in the samples of 2017 and 2018, but no residual effect of manure and cover crops was observed 2 years later on root-lesion nematodes in spring 2019. However, relatively lower values from treatments including forage pearl millet and pure stand sorghum sudangrass were observed (Table 5).

## 4. Discussion

### 4.1. Effect of Manure and Cover Crops on Total N Accumulation in Cover Crops and on Soil Nitrate Dynamics

It is a common practice in PEI to plow down red clover in fall to be able to seed potato as early as possible in the following spring but this has increased the risk of nitrate leaching over the winter and reduced N carried over to the subsequent crop [18,47,48,49]. Our results corroborated those findings, as red clover was associated with higher soil nitrate at the time of cover crop incorporation in comparison with other treatments. Nitrate measured overwinter using AEMs was higher for the treatment including red clover and forage sorghum followed by brown mustard, which was characterized by a lower C:N ratio in comparison with other treatments. Total N accumulated in red clover prior to cover crop incorporations was on average 88% higher than the treatments where grasses and legumes were mixed (alfalfa and orchardgrass mixture, ryegrass and common vetch and crimson clover mixture, sorghum sudangrass and verticillium-resistant alfalfa, and winter rye and hairy vetch mixture), and yet total tuber yields were comparable. Compared with red clover, mixing grasses with legumes allowed a decrease in nitrate susceptible to leaching without compromising potato yield, with alfalfa mixed with orchardgrass having the lowest numerical value of nitrate in fall at the time of cover crop incorporation.

### 4.2. Effects of Manure and Cover Crops on Potato Yield, Specific Gravity, and on Jelly End Incidence

Due to a low rainfall in July, total yield observed in this study was in the lower range than those reported in New Brunswick on different cultivars with average total yields ranging from 30 to 47 Mg·ha^−1^ over 2 years [50], and in Nova Scotia on Superior cultivar with yields up to 35 Mg·ha^−1^ [51]. However, yield values in these studies were comparable to those reported in Quebec on Russet Burbank, with an average total yield over 5 years ranging from 16 to 38 Mg·ha^−1^ [52]. Similar yield ranges were also reported in studies conducted by Zebarth et al. [53] in New Brunswick on different varieties, with yields ranging from 13 to 49 Mg·ha^−1^, and by Snowdon et al. [54] with average yields of 34.4 Mg·ha^−1^ in a study comparing different potato rotations. Manure applied during cover crop phase one year before potatoes significantly increased total and marketable yields, as demonstrated in other previous studies [12,14,15]. Nitrogen fertilizer application significantly increased total yield and reduced specific gravity. Specific gravity is a key parameter of potato processing quality and is related to tuber starch or solid content and thus chip yields. As a potato tuber matures, specific gravity increases proportionally to starch accumulation. Reduced potato specific gravity with increasing N rates was reported in previous studies [55,56,57], most likely because N application extends vegetative growth and can delay tuber initiation. Nitrogen effect was not significant on marketable yield but significantly increased jelly end incidence. Excluding tubers showing sign of jelly end resulted in lower marketable yield in comparison with total yield. 

The main causes of jelly end incidence are high temperature and moisture stress [58], which reflect the drought spell observed in 2018 during the potato phase, with rainfall in July being eight times lower than the 30-year average. We observed the percentage of jelly end to increase with N fertilizer application. Affected tubers have a translucent or glassy, soft and spongy end appearance, resulting in French fries that are darker in one end making them less desirable by consumers [58]. In addition, this can affect tuber quality during storage, forming jelly end rot. Under optimal moisture supply and temperature, the photosynthesis exceeds respiration, and sugars and starch are available to be translocated from leaves to tubers. However, when photosynthesis is reduced under moisture stress and high temperature, sugars and starch required for respiration may come from storage organs such as stems and tubers to support growth and recovery, which negatively impacts tuber bulking with increased jelly end formation [59,60]. Jelly end incidence was also attributed to a translocation of carbohydrates from the potato bottom end to the top end [61], and to a disruption of normal biochemical processes causing deterioration of the tuber bottom end [62]. Percent of jelly end may have increased with N fertilizer more than with no fertilizer application, because N fertilizer increases the tuber growth rate. The most sensitive period in developing jelly end was reported to happen during early tuber bulking [63,64,65,66], and short, intense periods of moisture stress and high temperature were reported to be more detrimental than extended periods of heat and moisture deficit [67].

### 4.3. Effects of Manure and Cover Crops on Soil Properties and on Soil N Supply Capacity

Soil degradation, including soil C depletion and decreased soil aggregation, were reported in potato-based systems [5]. Organic amendments are an effective means to build soil quality faster than cover crops. Compared to no manure application, manure incorporation increased total and marketable yield by 28% and 26%, respectively. In addition, there was a clear trend towards increased microbial respiration and SOC with manure application. This study demonstrated that moderate amounts of manure applied during the cover crop phase could be an effective way to increase potato yield, and corroborates previous studies [4,12,13,14,15]. 

Both cover crops and manure application may contribute to increased soil N supply capacity. Using a plant bioassay approach as total N uptake in unfertilized plot to reflect soil N supply [68], soil alone supplied 38–51 kg·N·ha^−1^ without manure application and 44–81 kg·N·ha^−1^ when manure was applied. Manure application increased the soil N supply by an average of 44%. This could increase soil N supply and decrease our reliance on N fertilizers. Increased efficiency in the use of N from manure during potato growth was reported in other studies [12,15,69]. Soil N supply estimated as total N accumulation in plots without N fertilizer was reported to be a valid index of plant available soil N supply to potatoes and to integrate climate and management practices [68].

The effects of cover crops or manure application were not significant on total organic matter, active carbon, earthworm abundance, and surface hardness. A longer period is needed to observe significant changes in soil health including soil organic matter [29]. The relatively low amount of manure applied once over 2 years combined with intensive and frequent soil tillage specifically during potato phase likely diluted the manure to a point where it was difficult to detect its effects. A flush of carbon dioxide after rewetting a dried soil following 24-h incubation increased by 27% on average in manured soils compared with unmanured ones and thus appeared to be more sensitive to reflecting manure application than the active carbon. Our results are aligned with a previously study [70]. Using 20 soil series with a broad range of organic matter contents from different sites, the latter authors reported that this index explained 97%, 86%, and 67% of the variation of cumulative C mineralization, soil microbial biomass, and net N mineralization, respectively [70]. Another study conducted in Texas reported a close relationship between this index and potentially mineralizable C and N and with microbial biomass C [71]. The flush of CO_2_ after rewetting soil was also reported to reflect organic matter decomposition rate in another study [72]. Our study suggests that a flush of CO_2_ following a short incubation was more sensitive than the active carbon in reflecting potential soil biological activity in response to manure application. 

### 4.4. Effects of Manure and Cover Crops on Population Density of Verticillium dahliae and Root-Lesion Nematodes

*V. dahliae* can infect over 400 plant species, including alfalfa, clover, rapeseed, and some other rotation crops [73,74]. Host specificity has been identified within the species. Strains were classified into several vegetative compatibility groups (VCG). Vegetative compatibility group 4 is virulent on potato and related Solanaceous species in North America [75]. A variety of DNA assays by qPCR has been developed and used to determine the population densities of soil-borne pathogens [76,77,78]. It was reported that DNA of fungi and nematodes became mostly undetectable within a week after the organisms had died, meaning that results of the assays closely approximated the DNA density in soil for living organisms [76]. In the present study, the DNA quantity of *V. dahliae* was estimated using qPCR based on species-specific primer and probe sequences targeting the *ef1α* gene, which could include all VCG strains present in the field. Therefore, the population density of living strains virulent to potatoes based on the DNA quantification could be overestimated. Rapeseed and sorghum sudangrass as cover crops were reported as significantly reducing population density of *V. dahliae* [79]. However, in the present study, no significant differences on population density of *V. dahliae* following cover crops were detected in fall of the rotation year, in spring of the potato year, or in spring following potato production. This difference could be attributable to different detection methods and soil types and other environmental conditions [79,80]. Manure effect on V. *dahliae* was short lived, as manure was associated with lower density in fall 2017, but the effect was not maintained and the opposite trend tended to be observed in fall 2018 and spring 2019. More trials on the effect of manure on V. *dahliae* are needed.

Red clover, a traditional rotation crop, and potato are favorable hosts of *V. dahliae* and root-lesion nematode (*Pratylenchus penetrans*), pathogens causing PED, and wireworm causing tuber-feeding damage [81]. Recent efforts have focused on identifying alternative potato rotational crops to red clover to break the disease cycle under potato phase. The effect of the cover crops on root-lesion nematode population was not determined in fall 2017 after crop residue incorporation and in spring 2018 prior to seeding potatoes. The residual effect of the cover crops after potato was not statistically significant in spring 2019. This corroborated results from other studies indicating that the root-lesion nematode population density after treatments would rebound after one cropping season [82,83]. Pearl millet and sorghum sudangrass were associated with lower numerical value of root-lesion nematodes following potato production and were associated with significantly lower soil nitrate in comparison to red clover while being associated with higher potato yield. Other previous studies have reported yield benefits following sorghum sudangrass [19,34] and lower root-lesion nematode with pearl millet [30,31,32,33,79]. 

## 5. Conclusions

Red clover was associated with higher soil nitrate at the time of cover crop incorporation, but this was not translated into increased potato yield. Pearl millet and sorghum sudangrass were associated with higher potato total yield and lower soil nitrate susceptible to leaching than red clover, and there was a trend toward lower numerical values of root-lesion nematode. Manure application significantly increased total and marketable yield. Manure application also increased soil N supply by an average of 44%. The effect of manure was not associated with increased cover crop biomasses, but trends toward increased yield and soil N supply following manure incorporation must be attributed to indirect effects such as increased soil moisture retention and/or enhanced soil microbial activity. Drier conditions over the potato growing season increased jelly end incidence when N fertilizer was applied. There was a trend of increased CO_2_ following a short soil incubation and increased SOC following manure application. Carbon dioxide following an incubation of rewetted air-dried soil could be a more sensitive index of management practice than active carbon as measured with permanganate oxidizable carbon. Manure applied on cover crops preceding potato, and high-residue cover crops such as pearl millet and sorghum sudangrass are promising means to enhance soil health and to increase potato yield. Results from this study were based on only one season of cover crops and one year of manure application and different results can be expected under different pedo-climatic conditions.

## Figures and Tables

**Table 1:** Effects of manure application and cover crop treatments on cover crop dry matter, carbon and nitrogen accumulations, and C:N ratio prior to cover crop incorporation.

Sources of Variation	Total Dry Matter	Total Carbon Accumulation	Total Nitrogen Accumulation	C:N Ratio
	Mg·ha^−1^	kg·ha^−1^		
Manure application				
With manure	4.3 a	1845.8 a	93.6 a	19.1 a
Without manure	4.3 a	1866.8 a	98.4 a	19.2 a
Standard error of the mean	0.22	93.7	4.8	0.42
Cover crop treatments				
Alfalfa and orchardgrass	3.7 c	1577.7 c	74.2 c	20.6 ab
Forage pearl millet	6.2 a	2664.9 a	112.9 b	22.3 a
Forage sorghum followed by brown mustard	3.9 c	1673.0 c	103.4 b	17 c
Red clover	4.9 b	2076.1 b	145.6 a	14.4 d
Ryegrass and common vetch and crimson clover	4.7 bc	2023.9 b	97.7 b	19.6 b
Sorghum sudangrass	4.7 bc	2059.2 b	90.9 bc	21.5 ab
Sorghum sudangrass and VR alfalfa	3.6 c	1588.7 c	67.7 c	21.6 ab
Winter rye and hairy vetch	2.7 d	1186.7 d	75.8 c	16.1 cd
Standard error of the mean	0.34	147.6	8.4	0.75
Analysis of variance				
Manure effect (M)	NS	NS	NS	NS
Treatment effect (T)	***	***	***	***
M × T	NS	NS	NS	NS

Values followed by different letters within a group are statistically different at 5% probability level. NS, not significant at 5% probability level; ***, significant at 0.001 probability level; VR, verticillium resistant. Carbon and nitrogen accumulations were obtained by multiplying the C and N concentration by the respective dry matter.

**Table 2:** Effects of manure application and cover crop treatments on soil nitrate during potato growing season without N fertilizer application.

Sources of Variations	May			June		July		August		September	
Sampling Depth (cm)											
	0–15	15–30		0–15	15–30	0–15	15–30	0–15	15–30	0–15	15–30
	mg NO_3_-N·kg^−1^										
Manure application											
With manure	5.1 a	6.6		10.8 a	14.5 a	12.2 a	16.6 a	5.3 a	8.3 a	5.8 a	6.6 a
Without manure	3.5 b	5.05		8.9 a	11.6 a	8.6 a	12.5 a	4.5 a	6.3 a	6.0 a	6.7 a
Standard error of the mean	0.43	0.53		0.67	0.83	0.82	0.98	0.58	0.97	0.56	0.72
Cover crop treatments											
		No manure	With manure								
Alfalfa and orchardgrass	5.1 a	4.9 bc	6.1 bc	10.7 a	13.7 a	11.5 a	15.4 a	6.2 a	8.6 a	6.6 a	5.9 a
Forage pearl millet	3.9 c	4.9 c	5.1 d	9.4 a	12.9 a	8.9 a	13.2 a	4.1 a	7.5 a	6.0 a	6.6 a
Forage sorghum followed by brown mustard	4.1 bc	6.1 b	7.4 ab	9.9 a	14.1 a	10.8 a	14.1 a	3.6 a	5.3 a	4.2 a	5.1 a
Red clover	5.1 ab	7.3 a	6.6 ab	10.6 a	13.0 a	9.4 a	13.0 a	5.4 a	7.3 a	7.2 a	9.4 a
Ryegrass and common vetch andcrimson clover	5.5 a	2.8 c	8.4 a	8.8 a	10.3 a	10.9 a	15.2 a	5.0 a	8.9 a	5.3 a	6.3 a
Sorghum sudangrass	3.2 c	4.4 bc	5.7 cd	8.6 a	12.6 a	10.0 a	15.1 a	5.1 a	8.0 a	6.5 a	7.1 a
Sorghum sudangrass and VR alfalfa	4.4 bc	3.9 bc	5.4 cd	10.2 a	13.6 a	9.4 a	14.0 a	4.5 a	5.9 a	5.9 a	6.3 a
Winter rye and hairy vetch	4.7 bc	5.4 bc	6.1 bc	10.6	13.7 a	12.3 a	16.7 a	4.9a	6.8 a	5.6 a	6.1 a
Standard error of the mean	0.55	1.063	1.063	1.087	1.34	1.6	1.91	0.82	1.37	0.98	0.99
Analysis of variance											
Manure effect (M)	*	NS		NS	NS	NS	NS	NS	NS	NS	NS
Treatment effect (T)	**	*		NS	NS	NS	NS	NS	NS	NS	NS
M × T	NS	*		NS	NS	NS	NS	NS	NS	NS	NS

Values followed by different letters within a group are statistically different at 5% probability level. NS, not significant at 5% probability level; *, **, significant at 0.05 and 0.01 probability levels, respectively; VR, verticillium resistant.

**Table 3:** Effects of manure, nitrogen applications, and cover crop treatments on potato total and marketable yield and specific gravity.

Sources of Variations	Total Yield	Marketable Yield	Specific Gravity
	Mg·ha^−1^		
Manure application			
With manure	30.3 a	24.5 a	1.0786 a
Without manure	23.6 b	18.1 b	1.0747 a
Standard error of the mean	1.34	1.71	0.001
Nitrogen application			
With N fertilizer	28.9 a	21.05 a	1.0732 b
Without N fertilizer	24.9 b	21.5 a	1.080 a
Standard error of the mean	1.21	1.40	0.001
Cover crop treatments			
Alfalfa and orchardgrass	27.2 ab	21.05 a	1.0775 a
Forage pearl millet	29.8 a	24.2 a	1.0788 a
Forage sorghum followed by brown mustard	29.9 a	24.1 a	1.0715 a
Red clover	24.5 b	18.8 c	1.0797 a
Ryegrass and common vetch and crimson clover	23.9 b	18.7 c	1.0764 a
Sorghum sudangrass	28.0 a	22.1 ab	1.0784 a
Sorghum sudangrass and VR alfalfa	24.8 b	20.5 bc	1.0779 a
Winter rye and hairy vetch	27.1 ab	20.6 bc	1.0770 a
Standard error of the mean	1.59	1.71	0.002
Analysis of variance			
Manure effect (M)	*	*	NS
Treatment effect (T)	**	**	NS
M × T	NS	NS	NS
N fertilizer (N)	***	NS	***
T × N	NS	NS	NS
M × N	NS	*	NS
M × T × N	NS	NS	NS

Values followed by different letters within a group are statistically different at 5% probability level. NS, not significant at 5% probability level; *, **, *** significant at 0.05, 0.01, and 0.001 probability levels, respectively; VR, verticillium resistant.

**Table 4:** Effects of manure, nitrogen fertilizer applications and cover crop treatments on permanganate oxidizable carbon, total earthworm (individual count) and surface hardness.

Sources of Variations	Permanganate Oxidazable Carbon	Total Earthworm (m^−2^)	Surface Hardness (PSI)
	(mg C kg^−1^)		
Manure application			
With manure	368.4 a	18.6 a	216.2 a
Without manure	372.4 a	12.3 a	203.1 a
Standard error of the mean	14.3	2.2	24.3
Cover crop treatments			
Alfalfa and orchardgrass	375.5 a	11 a	189.2 a
Forage pearl millet	378.1 a	14 a	190.3 a
Forage sorghum followed by brown mustard	359.8 a	14 a	190.0 a
Red clover	383.2 a	14 a	230.6 a
Ryegrass and common vetch and crimson clover	360.7 a	14 a	242.2 a
Sorghum sudangrass	362.1 a	22 a	220.6 a
Sorghum sudangrass and VR alfalfa	374.5 a	16 a	196.7 a
Winter rye and hairy vetch	369.6 a	18 a	217.5 a
Standard error of the mean	15.13	4.4	28.4
Analysis of variance			
Manure effect (M)	NS	NS	NS
Treatment effect (T)	NS	NS	NS
M × T	NS	NS	NS

Values followed by the same letters within a group are not statistically different at 5% probability level. NS, not significant at 5% probability level; VR, verticillium resistant.

**Table 5:** Effects of manure application and cover crop treatments on population densities of *Verticillium dahliae* and root-lesion nematodes.

Sampling Time	2017 Fall	2018 Spring	2019 Spring	
Sources of Variations	DNA (pg·g^−1^ Dry Soil)	DNA (pg·g^−1^ Dry Soil)	DNA (pg·g^−1^ Dry Soil)	Root-Lesion Nematodes (kg^−1^ Dry Soil)
Manure application				
With manure	37.5 a	26 a	112.3 a	3246.2 a
Without manure	49.1 b	22.4 a	93.7 a	2442.5 a
Standard error of the mean	3.9	3.1	23.4	633.5
Cover crop treatments				
Alfalfa and orchardgrass	36.9 a	24.2 a	83.9 a	2432.5 a
Forage pearl millet	53.4 a	28.9 a	68.5 a	1410 a
Forage sorghum followed by brown mustard	38.3 a	18.7 a	134.6 a	3390 a
Red clover	45.7 a	22.6 a	86.9 a	3177.5 a
Ryegrass and common vetch and crimson clover	34.4 a	18.4 a	57.9 a	3535 a
Sorghum sudangrass	45.2 a	29.7 a	99.3 a	2335 a
Sorghum sudangrass and VR alfalfa	46.4 a	22.8 a	181.9 a	3117.5 a
Winter rye and hairy vetch	46 a	28.1 a	110.9 a	3357.5 a
Standard error of the mean	7.8	6.3	46.8	858.5
Analysis of variance				
Manure (M)	*	NS	NS	NS
Treatment effect (T)	NS	NS	NS	NS
M × T	NS	NS	NS	NS

*, NS, significant and not significant at 5% probability level, respectively; VR, verticillium resistant. Values followed by the same letters within a group are not statistically different at 5% probability level.
